# The impact of mammographic screening on the subsequent breast cancer risk associated with biopsy-proven benign breast disease

**DOI:** 10.1038/s41523-021-00225-9

**Published:** 2021-03-05

**Authors:** Francisco Beca, Hannah Oh, Laura C. Collins, Rulla M. Tamimi, Stuart J. Schnitt

**Affiliations:** 1 Department of Pathology, Stanford University Medical School, Stanford, CA USA; 2 Interdisciplinary Program in Precision Public Health, College of Health Sciences, Korea University, Seoul, South Korea; 3 Department of Pathology, Beth Israel Deaconess Medical Center and Harvard Medical School, Boston, MA USA; 4 Channing Division of Network Medicine, Brigham and Women’s Hospital and Harvard Medical School, Boston, MA USA; 5 Department of Pathology, Brigham and Women’s Hospital and Harvard Medical School, Boston, MA USA; 6 Dana-Farber Cancer Institute, Boston, MA USA

**Keywords:** Risk factors, Medical research, Breast cancer

## Abstract

Data on the risk of breast cancer following a benign breast disease (BBD) diagnosis were derived predominantly from populations of women biopsied before the widespread use of mammographic screening and in whom these lesions were mostly incidental findings. Whether or not similar risk associations are seen when these lesions are detected in mammographically screened populations is unknown. To address this, we examined the variation in BBD and breast cancer risk associations by the calendar time of BBD diagnosis (pre- vs. post-mammography era [before vs. 1985 and after]) in a nested case–control study within the Nurses’ Health Study (NHS) and NHSII BBD subcohort (488 cases; 1908 controls). We performed logistic regression analysis, adjusting for matching factors and potential confounders, to estimate odds ratio (ORs) and 95% confidence interval (CI) for the association between BBD subtype (non-proliferative, proliferative without atypia, proliferative with atypical hyperplasia (AH)) and subsequent breast cancer risk. When compared with non-proliferative lesions, both proliferative lesions without atypia (PWA) and AHs were associated with similar levels of risk in the pre-mammographic (pre) and post-mammographic (post) time periods (PWA: OR [95% CI] = 1.73 [1.27, 2.36] pre vs. 1.12 [0.73, 1.74] post; AH: 4.41 [2.90, 6.70] pre vs. 3.69 [2.21, 6.15] post). The interaction by mammography era was not statistically significant (*p*-interaction = 0.47). These results suggest that the risk associations reported for BBD subtypes in the pre-mammography era remain valid for BBD detected after the widespread implementation of mammographic screening.

## Introduction

Benign breast disease (BBD) is a term that encompasses a heterogeneous group of lesions with different levels of subsequent breast cancer risk. To date, most epidemiologic studies, including studies from our group, have shown that proliferative lesions without atypia are associated with a 1.5- to 2-fold increase in breast cancer risk, whereas atypical hyperplasias are associated with a 4–5-fold increase in risk^1–4^. However, these data were derived from populations of women who, for the most part, were biopsied before the widespread use of screening mammography and in whom these lesions were incidental findings in the context of a clinically symptomatic lesion, most often a palpable mass, that was surgically excised^5^. It is unknown whether the BBD risk associations based on biopsies performed in the pre-mammography era^1–4^ are applicable to the BBDs detected in biopsies performed for a non-palpable lesion detected by screening mammography. This is an important clinical question that could have a significant impact on individual patient counseling, risk estimation models, and design and evaluation of preventive interventions.

In this study, we examined whether the associations of BBD subtype (non-proliferative lesions, proliferative lesions without atypia, atypical hyperplasia) with breast cancer risk were similar between the pre- and post-mammography era (before vs. 1985 and after). We also examined whether the associations vary by BBD detection method (mammogram vs. self-exam/physician’s exam). For these analyses, we used a nested case–control study within the Nurses’ Health Study (NHS) and NHSII BBD subcohort. Because mammographic screening was widely implemented in the United States in the mid-1980, we used 1985 as the surrogate year for defining pre- vs. post-mammography era in primary analyses.

## Results

### Population characteristics

Participant characteristics of 488 cases and 1908 controls in the pre- and post-mammography era are presented in Table 1. The mean age at BBD biopsy among controls was 41.0 years in pre-mammography era and 49.8 years in the post-mammography period. Compared to women with BBDs detected during pre-mammography era, those with BBDs detected during post-mammography era were more likely to have atypical hyperplasia, higher adult body mass index (BMI), and higher levels of physical activity. They were also less likely to be current smokers. Among controls, 43.5% of BBDs diagnosed during the post-mammography era were detected on mammograms compared with 8.7% during pre-mammography era.

**Table 1:** Participant characteristics according to case–control status and pre- (before 1985) vs. post-mammography era (1985 and after) of BBD diagnosis.

	*N* (%) or mean (SD)			
	Pre-mammography era (before 1985) *n* = 1469		Post-mammography era (1985 and after) *n* = 927	
	Cases (*n* = 302)	Controls (*n* = 1167)	Cases (*n* = 186)	Controls (*n* = 741)
Age at benign biopsy^a^ (years)	40.7 (8.9)	41.0 (9.0)	49.9 (9.5)	49.8 (9.9)
Menopausal status^a^				
Premenopausal (%)	246 (81.5)	922 (79.0)	95 (51.1)	377 (50.9)
Postmenopausal (%)	37 (12.3)	154 (13.2)	71 (38.2)	308 (41.6)
Unknown or missing (%)	19 (6.3)	91 (7.8)	20 (10.8)	56 (7.6)
BBD subtype^a^				
Non-proliferative (%)	72 (23.8)	474 (40.6)	36 (19.4)	206 (27.8)
Proliferative without atypia (%)	163 (54.0)	590 (50.6)	93 (50.0)	446 (60.2)
Atypical hyperplasia (%)	67 (22.2)	103 (8.8)	57 (30.7)	89 (12.0)
Method of BBD detection^a^				
Finding on mammography (%)	19 (6.3)	102 (8.7)	77 (41.4)	322 (43.5)
Self-exam or physical exam^b^ (%)	243 (80.5)	952 (81.6)	80 (43.0)	330 (44.5)
Other (%)	40 (13.3)	113 (9.7)	29 (15.6)	89 (12.0)
Height (in.)	64.6 (2.3)	64.5 (2.5)	64.6 (2.2)	64.8 (2.6)
Average body size at age 5–10 years				
Levels 1–2 (%)	183 (60.6)	671 (57.5)	94 (50.5)	385 (52.0)
Levels 2.5–4 (%)	66 (21.9)	251 (21.5)	56 (30.1)	215 (29.0)
Level ≥4.5 (%)	12 (4.0)	85 (7.3)	10 (5.4)	47 (6.3)
Missing (%)	41 (13.6)	160 (13.7)	26 (14.0)	94 (12.7)
Adult BMI at benign biopsy^a^ (kg/m^2^)	23.3 (3.0)	23.6 (3.7)	24.4 (4.1)	24.9 (4.7)
Age at menarche (years)	12.6 (1.4)	12.6 (1.4)	12.3 (1.3)	12.6 (1.4)
Age at first birth^a^				
Nulliparous (%)	20 (6.6)	75 (6.4)	18 (9.7)	71 (9.6)
<25 years (%)	120 (39.7)	539 (46.2)	90 (48.4)	343 (46.3)
25–29 years (%)	113 (37.4)	387 (33.2)	54 (29.0)	262 (35.4)
≥30 years (%)	30 (9.9)	103 (8.8)	19 (10.2)	59 (8.0)
Missing (%)	19 (6.3)	63 (5.4)	5 (2.7)	6 (0.8)
Current smoking^a^ (%)	65 (21.5)	243 (20.8)	31 (16.7)	93 (12.6)
Alcohol intake^a^ (g/day)	5.4 (7.1)	6.0 (9.2)	6.2 (10.9)	4.9 (7.4)
Physical activity level^a^ (MET-h/week)	13.4 (12.9)	14.8 (17.7)	16.0 (25.5)	16.4 (18.6)
Family history of breast cancer^a^ (%)	52 (17.2)	141 (12.1)	35 (18.8)	86 (11.6)

Values are means (SD) for continuous variables; *N* (percentages) for categorical variables. Values of polytomous variables may not sum to 100% due to rounding.

*BBD* benign breast disease, *BMI* body mass index, *MET* metabolic equivalent task.

^a^At benign breast biopsy.

^b^Includes benign breast disease detected on both self-exam/physical exam and mammography because we assume these benign breast diseases were first found on self-exam or physician’s physical exam (palpable mass).

### BBD subtype and breast cancer risk

In all women, proliferative lesions without atypia and atypical hyperplasia were associated with a 1.50-fold (95% confidence interval [CI] = 1.17–1.93) and 4.24-fold (95% CI = 3.08–5.83) higher breast cancer risk, respectively, compared with non-proliferative lesions (Table 2). Similar associations were found with vs. without adjustment for potential confounders. When stratified by mammography era, proliferative lesions without atypia were associated with a 1.73-fold higher risk of breast cancer (95% CI = 1.27–2.36), and atypical hyperplasia was associated with a 4.41-fold higher risk (95% CI = 2.90–6.70) compared with non-proliferative lesions during the pre-mammography era (Table 2). The risk associations were similar during the post-mammography era (proliferative lesions without atypia vs. non-proliferative: odds ratio [OR] = 1.12, 95% CI = 0.73–1.74; atypical hyperplasia vs. non-proliferative: OR = 3.69, 95% CI = 2.21–6.15) and the interaction by mammography era was not statistically significant (*p*-interaction = 0.47). When BBD subtype was further categorized into atypical ductal and lobular hyperplasia, the highest risk was observed with atypical lobular hyperplasia (pre-mammography era: OR = 6.16, 95% CI = 3.43–11.1; post-mammography era: OR = 6.17, 95% CI = 3.23–11.8), followed by atypical ductal hyperplasia (pre-mammography era: OR = 3.58, 95% CI = 2.18–5.88; post-mammography era: OR = 2.55, 95% CI = 1.38–4.71) in both pre- and post-mammography era (*p*-interaction=0.63) (Supplementary Table 1). When stratified by menopausal status, the association with atypical hyperplasia appeared stronger in postmenopausal women than in premenopausal women, but the interaction was not statistically significant in both the pre- (*p*-interaction=0.66) and post-mammography era (*p*-interaction=0.60) (Supplementary Table 2).

**Table 2:** OR and 95% CIs for the associations between benign breast disease subtypes and breast cancer risk, stratified by pre- (before 1985) vs. post-mammography era (1985 and after) of BBD diagnosis.

Calendar year of benign breast disease detection	Benign breast disease subtype	Cases/controls	Model 1^a^ OR (95% CI)	Model 2^b^ OR (95% CI)
All BBDs	Non-proliferative lesion	108/680	1.0 (Ref.)	1.0 (Ref.)
	Proliferative lesion without atypia	256/1036	1.57 (1.23, 2.01)	1.50 (1.17, 1.93)
	Atypical hyperplasia	124/192	4.39 (3.20, 6.01)	4.24 (3.08, 5.83)
Pre-mammography era (<1985) *n* = 1469	Non-proliferative lesion	72/474	1.0 (Ref.)	1.0 (Ref.)
	Proliferative lesion without atypia	163/590	1.82 (1.35, 2.47)	1.73 (1.27, 2.36)
	Atypical hyperplasia	67/103	4.68 (3.11, 7.03)	4.41 (2.90, 6.70)
Post-mammography era (≥1985) *n* = 927	Non-proliferative lesion	36/206	1.0 (Ref.)	1.0 (Ref.)
	Proliferative lesion without atypia	93/446	1.12 (0.74, 1.72)	1.12 (0.73, 1.74)
	Atypical hyperplasia	57/89	3.56 (2.16, 5.85)	3.69 (2.21, 6.15)
*p*-interaction by mammography era			0.27	0.47

*OR* odds ratios, *CIs* confidence intervals, *BBD* benign breast disease.

^a^Model 1 includes matching factors (age at index date, time between benign biopsy to breast cancer diagnosis or index date, calendar year of benign breast biopsy).

^b^Model 2 includes matching factors, average body fatness at ages 5–10 years (levels 1–2, 2.5–4, ≥4.5, missing), adult BMI at benign biopsy (kg/m^2^, continuous), parous/age at first birth (nulliparous, <25 years, 25–29 years, ≥30 years, missing), menopausal status at benign breast biopsy (premenopausal, postmenopausal, dubious/missing), current smoking (yes/no), alcohol intake (g/day, continuous), physical activity (MET-h/week, continuous), family history of breast cancer (yes/no). Additional adjustment for height (inches, continuous) and age at menarche (years, continuous) did not change the results, and thus were not included in the final model.

Since many of the lesions in the pre-mammography era were biopsied due to the presence of a clinically symptomatic lesion, we also stratified the analysis by the method of BBD detection. In this analysis, the risk associations were also similar between the stratum of BBD found on self-exam or physician’s exam (proliferative without atypia vs. non-proliferative: OR = 1.49, 95% CI = 1.11–2.01; atypical hyperplasia vs. non-proliferative: OR = 3.98, 95% CI = 2.68–5.92) and the stratum of BBDs found on mammography (proliferative without atypia vs. non-proliferative: OR = 1.45, 95% CI = 0.71–2.98; atypical hyperplasia vs. non-proliferative: OR = 5.12, 95% CI = 2.36–11.1) and the interaction was not statistically significant (*p*-interaction=0.11) (Table 3).

**Table 3:** ORs and 95% CIs for the associations between benign breast disease subtypes and breast cancer risk, stratified by the method of BBD detection.

Method of benign breast disease detection	Benign breast disease subtype	Cases/controls	Model 1^a^ OR (95% CI)	Model 2^b^ OR (95% CI)
First found on mammography (*n* = 520)	Non-proliferative lesion	12/103	1.0 (Ref.)	1.0 (Ref.)
	Proliferative lesion without atypia	45/254	1.54 (0.77, 3.09)	1.45 (0.71, 2.98)
	Atypical hyperplasia	39/67	5.20 (2.47, 11.0)	5.12 (2.36, 11.1)
Finding on self-exam or physician’s physical exam^c^ (*n* = 1605)	Non-proliferative lesion	82/501	1.0 (Ref.)	1.0 (Ref.)
	Proliferative lesion without atypia	170/669	1.59 (1.19, 2.12)	1.49 (1.11, 2.01)
	Atypical hyperplasia	71/112	4.22 (2.86, 6.22)	3.98 (2.68, 5.92)
Other (*n* = 271)	Non-proliferative lesion	14/76	1.0 (Ref.)	1.0 (Ref.)
	Proliferative lesion without atypia	41/113	1.82 (0.91, 3.66)	1.92 (0.90, 4.11)
	Atypical hyperplasia	14/13	5.37 (1.91, 15.1)	6.46 (2.04, 20.5)
*p*-interaction			0.56	0.11

Note: The method of BBD detection was categorized regardless of BBD diagnosis year (pre- or post-mammography era).

*OR* odds ratios, *CIs* confidence intervals, *BBD* benign breast disease.

^a^Model 1 includes matching factors (age at index date, time between benign biopsy to breast cancer diagnosis or index date, calendar year of benign breast biopsy).

^b^Model 2 includes matching factors, average body fatness at ages 5–10 years (levels 1–2, 2.5–4, ≥4.5, missing), adult BMI at benign biopsy (kg/m^2^, continuous), parous/age at first birth (nulliparous, <25 years, 25–29 years, ≥30 years, missing), menopausal status at benign breast biopsy (premenopausal, postmenopausal, dubious/missing), current smoking (yes/no), alcohol intake (g/day, continuous), physical activity (MET-h/week, continuous), family history of breast cancer (yes/no). Additional adjustment for height (inches, continuous) and age at menarche (years, continuous) did not change the results, and thus were not included in the final model.

^c^Includes benign breast disease detected on both self-exam/physical exam and mammography because we assume these benign breast diseases were first found on self-exam or physician’s physical exam.

### Sensitivity analyses

In a sensitivity analysis, the results did not change when 1980 was used to define the start of the mammography era (Supplementary Table 3). Because the results could have been influenced by the shorter follow-up time in the post-mammography era, we also excluded individuals with follow-up time >13 years (the maximum follow-up time in the post-mammography era) in another sensitivity analysis and results were similar (Supplementary Table 4).

## Discussion

Breast cancer risks associated with BBD subtypes were reported by studies that followed up large cohorts of women undergoing breast biopsies with different baseline characteristics and indications for biopsies, possibly reducing the ability to generalize these results to women undergoing breast biopsies for abnormalities detected by screening mammography. This observation not only has potential consequences for individual patient counseling but could affect some of today’s best-known breast cancer risk prediction models that use features like the number of biopsies and BBD subtype^6–11^. While these differences have been recognized in terms of breast cancer risk development models^5,6^, we investigated in this study whether the previously estimated BBD–cancer risk associations are still valid after the widespread introduction of screening mammography. In the current study, we found similar BBD–breast cancer risk associations from BBDs detected in the pre- vs. post-mammography era. Consistent with this, we also observed similar associations from BBDs first detected on self-exam or physician’s exam vs. BBDs detected on mammography. Our estimated associations were also similar to those previously reported in other studies from pre-mammography era utilizing distinct cohorts^1,5,12^. However, in contrast to a prior report from this cohort^13^, in the current study we found no significant differences in associations when stratifying by menopausal status particularly in the post-mammography era. In a previous iteration of this cohort, with a smaller sample size and shorter follow-up time, we had observed a stronger association with atypical hyperplasia in premenopausal (vs. postmenopausal) women^13^. This observation was not confirmed in this study in either the pre- or post-mammography era.

In this study, the use of biopsy-confirmed BBDs, the use of prospective study design, and the careful adjustment for potential confounders are important strengths. While we cannot exclude the existence of small significant differences, we were not able to detect these, given our limited sample size; if these existed, they would probably be clinically insignificant. Although sample size may be limited for stratified analyses, this study is still one of the largest studies to date that examined the BBD–breast cancer risk relationship. Our previous study published in 2007 included 395 cases and 1610 controls^3^, whereas this study includes 488 cases and 1908 controls with biopsy-confirmed BBDs. Although the exact year that should be used for the start of widespread mammography is difficult to determine, we confirmed 77% of NHS/NHSII women aged ≥40 years received mammography in 1988/1989. A proportion of BBDs first detected on mammogram was also higher in post-mammography era (1985 and after) compared with pre-mammography era (44 vs. 9%). Further, it is generally accepted that widespread access to mammographic screening happened in the United States in the mid-1980s, and, therefore, we believe 1985 is an appropriate surrogate^14–16^. It should also be noted that we had sparse information on biopsy method and thus were not able to determine the prevalence of core needle biopsies. This fact made stratified analyses by core needle biopsy vs. surgical biopsy impossible. While such an analysis would have been informative and potentially more generalizable to contemporary practice, it was not until the late 1990s/early 2000s that core needle biopsy became the principal method for the diagnosis of non-palpable breast lesions. As the NHS and NHSII are ongoing prospective studies, it may be possible in the future to confirm our results by including more recent data from patients with image-directed core-biopsied lesions. Nevertheless, we believe this study still provides important insights since it is the first study to date that has compared the BBD–breast cancer risk associations before and after the widespread implementation of screening mammography.

In conclusion, our data suggest that the magnitude of risk associated with BBD subtypes initially reported from women biopsied in the pre-mammography era remains valid for BBD detected after the widespread use of mammography. Given the limited information available on the type of biopsy performed, further studies of patients in which BBDs were detected by core-needle biopsy are needed to confirm our results.

## Methods

### Study subjects

This study is a nested case–control study within the NHS and NHS II cohorts with biopsy-confirmed BBD. The NHS is an ongoing prospective cohort study that began in 1976, when 121,700 female registered nurses between the ages of 30 and 55 years completed a mailed questionnaire. The NHS II consists of 116,609 female registered nurses who were between the ages of 25 and 42 years when the study began in 1989. In both cohorts, participants have been followed via biennial questionnaires that provide information on lifestyle factors (BMI, physical activity, smoking, and alcohol use), family history of disease, reproductive history, and incident disease^17,18^. Cumulative response rates for both cohorts were >90% and were similar among women regardless of their BBD diagnosis.

Details on the BBD diagnosis reporting on the questionnaires have been previously described^2,19^. Briefly, cases were women with biopsy-confirmed BBD who reported a subsequent diagnosis of breast cancer following their BBD diagnosis. Cases (invasive, in situ) were diagnosed between 1976 and 1998 for the NHS and between 1989 and 1999 for the NHS II. Self-reported breast cancers were confirmed by review of medical records. To reduce potential reverse causation, women were excluded if they had evidence of in situ or invasive carcinoma at biopsy or reported a diagnosis of breast cancer within 6 months of their BBD biopsy. There was a mean of 10 years (SD = 6.93) between BBD biopsy and cancer diagnosis. Eligible controls were women who completed the questionnaire in the same year that the breast cancer case was reported and had a previous diagnosis of biopsy-confirmed BBD but were free from breast cancer at the time of the case was diagnosed (index date; date of which the case was diagnosed). Up to four controls were selected for each breast cancer case, matched on year of birth and year of BBD biopsy. Women were excluded if they had unknown BBD lesion type. A total of 488 cases and 1908 controls, in whom the original slides had been reviewed, were eligible for this study. Due to considerable missing information on the laterality of the carcinoma in the cases, this information was not considered in the analysis. The study protocol was approved by the institutional review boards of the Brigham and Women’s Hospital and Harvard T.H. Chan School of Public Health, and those of participating registries as required. Completion of the self-administered questionnaire was presumed to imply informed consent. This study complies with the STROBE (Strengthening the Reporting of Observational Studies in Epidemiology) guidelines^20^.

### Benign breast biopsy specimens

Eligible cases and controls were contacted for permission to obtain their BBD pathology records and biopsy specimens, and specimens were then obtained from hospital pathology departments when possible (as detailed in refs. ^2,3,21^). The ability to obtain biopsy blocks did not significantly differ by case and control status. Two study pathologists independently reviewed the hematoxylin- and eosin-stained sections from the benign breast biopsy blocks and completed detailed worksheets on the subtype of BBD lesion (i.e., non-proliferative, proliferative without atypia, atypical ductal hyperplasia, atypical lobular hyperplasia) in a blinded manner. The details of this nested case–control study and the BBD assessment have been described previously^2,3,19,22^.

### Mode of BBD diagnosis

Women reported the year of BBD diagnosis and how they found BBD (finding on mammography, self-examination, physician’s examination, cyst aspiration before biopsy, other). We considered BBDs detected on both self-exam/physical exam and mammography as first found on self-exam/physical activity (palpable mass). We categorized women into those who had a BBD diagnosis in the pre- vs. post-mammography era. In primary analysis, we used 1985 as the cutpoint calendar year for defining pre- vs. post-mammography era because mammography screening was widely implemented in the United States. in the mid-1980. In the sensitivity analysis, we repeated the analyses using 1980 as the cutpoint calendar year for defining mammography era. Starting in 1988 in the NHS and 1989 in the NHSII, biennial questionnaires asked questions on whether women received mammogram and the year since the last mammogram. In the NHS, 77% of women have ever had any mammogram in 1988 (with an unknown proportion for screening mammogram) and, among these women, 56% had the most recent mammogram within 1 year. In the NHSII, 77% of women at age ≥40 years have ever had any mammogram in 1989 and, among these women, 79% had the routine screening and 46% had the most recent mammogram within 1 year. In 1990–1991, 76% of NHS participants and 70% of NHSII participants had a screening mammogram.

### Statistical analysis

To avoid losing data due to incomplete matched sets (benign biopsy blocks were not available for some cases and controls), we performed unconditional logistic regression analysis, adjusting for matching factors, to estimate ORs and 95% CIs for the association between BBD subtype (non-proliferative, proliferative without atypia, proliferative with atypical hyperplasia) and subsequent breast cancer risk. Multivariable models included matching factors (age at diagnosis [for cases] or at index date [for controls], time between biopsy to breast cancer diagnosis or index date, calendar year of benign breast biopsy) and potential confounders (average body fatness at ages 5–10 years, adult BMI at benign biopsy, age at first birth, menopausal status at benign breast biopsy, current smoking, alcohol intake, physical activity, family history of breast cancer). In the secondary analysis, we further categorized proliferative lesions with atypical hyperplasia into atypical ductal hyperplasia and atypical lobular hyperplasia. To assess the variation in associations by the mode of BBD diagnosis, we stratified the analyses by the calendar time of BBD diagnosis (pre- vs. post-mammography era [before vs. 1985 and after]) and the methods of BBD detection (mammogram vs. self-exam/physician’s exam vs. other). In the sensitivity analysis, we repeated the analyses using 1980 as the cutpoint calendar year for mammography era. Because results could be influenced by the different length of follow-up time in the pre- vs. post-mammography era, we also excluded individuals with follow-up time >13 years (the maximum follow-up time in the post-mammography era) in the sensitivity analysis. Interaction tests were performed using the likelihood ratio test for product terms.

All statistical tests were two-sided with 5% type I error. Analyses were conducted with SAS version 9 (version 9.2, SAS Institute, Cary, NC, USA).

### Reporting summary

Further information on experimental design is available in the Nature Research Reporting Summary linked to this paper.

## Supplementary information

Supplementary Material

Reporting Summary Checklist

## Data Availability

The data generated and analyzed during this study are described in the following metadata record: 10.6084/m9.figshare.13220867^23^. The data that support the findings of this study are available from the Nurses’ Health Studies but restrictions apply to the availability of these data, and so they are not publicly available. However, the data are available from the corresponding author upon reasonable request, and with the permission of Nurses’ Health Studies External Advisory Board. Additional data sharing information and policy details can be accessed at http://www.nurseshealthstudy.org/researchers.
