# Supplementary material for: The impact of mammographic screening on the subsequent breast cancer risk associated with biopsy-proven benign breast disease
Source: NPJ Breast Cancer. 2021 Mar 5;7:23. doi: 10.1038/s41523-021-00225-9 (PMC7935945; doi:10.1038/s41523-021-00225-9)
Supplement: Supplementary file 1 — Supplementary Material [file 41523_2021_225_MOESM1_ESM.pdf]

Article

The impact of mammographic screening on the subsequent breast cancer risk associated with biopsy-proven benign breast disease

Beca F et al.,

Supplementary Material:

Supplementary Tables 1-4

Supplementary Tables

Supplementary Table 1. Odds ratios (OR) and 95% confidence intervals (CIs) for the associations of non-proliferative, proliferative, atypical ductal hyperplasia, and atypical lobular hyperplasia with breast cancer risk, stratified by pre- (before 1985) vs. post-mammography era (1985 and after)

Calendar year of benign breast disease detection	Benign breast disease subtype	Cases/ Controls	Model 1[a]	Model 2[b]
			OR (95% CI)	OR (95% CI)
**Pre-mammography era (<1985) N=1469**	**Non-proliferative lesion**	72/474	1.0 (Ref)	1.0 (Ref)
	**Proliferative lesion without atypia**	163/590	**1.83 (1.35, 2.48)**	**1.74 (1.27, 2.37)**
	**Atypical ductal hyperplasia**	36/71	**3.66 (2.26, 5.92)**	**3.58 (2.18, 5.88)**
	**Atypical lobular hyperplasia**	30/31	**6.86 (3.88, 12.1)**	**6.16 (3.43, 11.1)**
**Post-mammography era (≥1985) N=927**	**Non-proliferative lesion**	36/206	1.0 (Ref)	1.0 (Ref)
	**Proliferative lesion without atypia**	93/446	1.13 (0.74, 1.73)	1.13 (0.73, 1.75)
	**Atypical ductal hyperplasia**	25/58	**2.48 (1.36, 4.51)**	**2.55 (1.38, 4.71)**
	**Atypical lobular hyperplasia**	30/32	**5.90 (3.15, 11.1)**	**6.17 (3.23, 11.8)**
**p-interaction**			0.42	0.63

[a] Model 1 includes matching factors (age at index date, time between benign biopsy to breast cancer diagnosis or index date, calendar year of benign breast biopsy)

[b] Model 2 includes matching factors, average body fatness at ages 5-10 years (level 1-2, 2.5-4, ≥4.5, missing), adult BMI at benign biopsy (kg/m^2, continuous), parous/age at first birth (nulliparous, <25 years, 25-29 years, ≥30 years, missing), menopausal status at benign breast biopsy (premenopausal, postmenopausal, dubious/missing), current smoking (yes/no), alcohol intake (g/d, continuous), physical activity (MET-hr/wk, continuous), family history of breast cancer (yes/no)

Supplementary Table 2.Odds ratios (OR) and 95% confidence intervals (CIs) for the associations between benign breast disease subtypes and breast cancer risk, stratified by menopausal status in the pre- (before 1985) and post-mammography (1985 and after) era of benign breast disease (BBD) diagnosis

Calendar year of benign breast disease detection	Benign breast disease subtype	Cases/ Controls	Model 1[a]	Model 2[b]
			OR (95% CI)	OR (95% CI)
**Pre-mammography era (<1985) N=1359**				
**Premenopausal women (N=1168)**	**Non-proliferative lesion**	64/378	1.0 (Ref)	1.0 (Ref)
	**Proliferative lesion without atypia**	131/470	**1.66 (1.19, 2.31)**	**1.58 (1.13, 2.22)**
	**Atypical hyperplasia**	51/74	**4.42 (2.80, 6.99)**	**4.44 (2.77, 7.11)**
**Postmenopausal women (N=191)**	**Non-proliferative lesion**	5/57	1.0 (Ref)	1.0 (Ref)
	**Proliferative lesion without atypia**	21/76	**3.60 (1.22, 10.7)**	**5.32 (1.31, 21.7)**
	**Atypical hyperplasia**	11/21	**6.24 (1.86, 21.0)**	**8.68 (1.99, 37.9)**
**p-interaction by menopausal status**			0.53	0.66
**Post-mammography era (≥1985) N=472**				
**Premenopausal women (N=472)**	**Non-proliferative lesion**	20/100	1.0 (Ref)	1.0 (Ref)
	**Proliferative lesion without atypia**	51/247	0.92 (0.51, 1.65)	0.88 (0.48, 1.65)
	**Atypical hyperplasia**	24/30	**3.68 (1.71, 7.89)**	**3.22 (1.44, 7.22)**
**Postmenopausal women (N=379)**	**Non-proliferative lesion**	13/88	1.0 (Ref)	1.0 (Ref)
	**Proliferative lesion without atypia**	33/168	1.30 (0.65, 2.62)	1.37 (0.66, 2.84)
	**Atypical hyperplasia**	25/52	**3.29 (1.54, 7.03)**	**4.33 (1.93, 9.73)**
**p-interaction by menopausal status**			0.57	0.60

[a] Model 1 includes matching factors (age at index date, time between benign biopsy to breast cancer diagnosis or index date, calendar year of benign breast biopsy)

[b] Model 2 includes matching factors, average body fatness at ages 5-10 years (level 1-2, 2.5-4, ≥4.5, missing), adult BMI at benign biopsy (kg/m^2, continuous), parous/age at first birth (nulliparous, <25 years, 25-29 years, ≥30 years, missing), menopausal status at benign breast biopsy (premenopausal, postmenopausal, dubious/missing), current smoking (yes/no), alcohol intake (g/d, continuous), physical activity (MET-hr/wk, continuous), family history of breast cancer (yes/no). Additional adjustment for height (inches, continuous) and age at menarche (years, continuous) did not change the results, and thus were not included in the final model.

Supplementary Table 3 Odds ratios (OR) and 95% confidence intervals (CIs) for the associations between benign breast disease subtypes and breast cancer risk, stratified by pre- (before 1980) vs. post-mammography era (1980 and after) of BBD diagnosis

Calendar year of benign breast disease detection	Benign breast disease subtype	Cases/ Controls	Model 1[a]	Model 2[b]
			OR (95% CI)	OR (95% CI)
**Pre-mammography era (<1980) N=1063**	**Non-proliferative lesion**	56/349	1.0 (Ref)	1.0 (Ref)
	**Proliferative lesion without atypia**	118/433	**1.72 (1.22, 2.45)**	**1.64 (1.14, 2.34)**
	**Atypical hyperplasia**	42/65	**4.27 (2.61, 6.97)**	**3.92 (2.37, 6.48)**
**Post-mammography era (≥1980) N=1333**	**Non-proliferative lesion**	52/331	1.0 (Ref)	1.0 (Ref)
	**Proliferative lesion without atypia**	138/603	**1.42 (1.00, 2.02)**	1.37 (0.96, 1.96)
	**Atypical hyperplasia**	82/127	**4.32 (2.85, 6.55)**	**4.22 (2.77, 6.45)**
**p-interaction**			0.73	0.75

[a] Model 1 includes matching factors (age at index date, time between benign biopsy to breast cancer diagnosis or index date, calendar year of benign breast biopsy)

[b] Model 2 includes matching factors, average body fatness at ages 5-10 years (level 1-2, 2.5-4, ≥4.5, missing), adult BMI at benign biopsy (kg/m^2, continuous), parous/age at first birth (nulliparous, <25 years, 25-29 years, ≥30 years, missing), menopausal status at benign breast biopsy (premenopausal, postmenopausal, dubious/missing), current smoking (yes/no), alcohol intake (g/d, continuous), physical activity (MET-hr/wk, continuous), family history of breast cancer (yes/no)

Supplementary Table 4 Odds ratios (OR) and 95% confidence intervals (CIs) for the associations between benign breast disease subtypes and breast cancer risk, stratified by pre- (before 1985) vs. post-mammography era (1985 and after), after excluding individuals with follow-up time >13 years

Calendar year of benign breast disease detection	Benign breast disease subtype	Cases/ Controls	Model 1[a]	Model 2[b]
			OR (95% CI)	OR (95% CI)
**Pre-mammography era (<1985) N=823**	**Non-proliferative lesion**	36/252	1.0 (Ref)	1.0 (Ref)
	**Proliferative lesion without atypia**	89/321	**1.89 (1.24, 2.89)**	**1.70 (1.10, 2.63)**
	**Atypical hyperplasia**	45/80	**4.22 (2.52, 7.07)**	**4.03 (2.35, 6.91)**
**Post-mammography era (≥1985) N=926**	**Non-proliferative lesion**	36/205	1.0 (Ref)	1.0 (Ref)
	**Proliferative lesion without atypia**	93/446	1.12 (0.73, 1.71)	1.11 (0.72, 1.72)
	**Atypical hyperplasia**	57/89	**3.54 (2.15, 5.82)**	**3.66 (2.19, 6.11)**
**p-interaction**			0.23	0.34

[a] Model 1 includes matching factors (age at index date, time between benign biopsy to breast cancer diagnosis or index date, calendar year of benign breast biopsy)
[b] Model 2 includes matching factors, average body fatness at ages 5-10 years (level 1-2, 2.5-4, ≥4.5, missing), adult BMI at benign biopsy (kg/m^2, continuous), parous/age at first birth (nulliparous, <25 years, 25-29 years, ≥30 years, missing), menopausal status at benign breast biopsy (premenopausal, postmenopausal, dubious/missing), current smoking (yes/no), alcohol intake (g/d, continuous), physical activity (MET-hr/wk, continuous), family history of breast cancer (yes/no)
