# Supplementary material for: The impact of mammographic screening on the subsequent breast cancer risk associated with biopsy-proven benign breast disease
Source: NPJ Breast Cancer. 2021 Mar 5;7:23. doi: 10.1038/s41523-021-00225-9 (PMC7935945; doi:10.1038/s41523-021-00225-9)
Supplement: Supplementary file 2 — Reporting Summary Checklist [file 41523_2021_225_MOESM2_ESM.pdf]

nature research

Corresponding author(s): Stuart J Schnitt

Last updated by author(s): Sep 13, 2020

# Reporting Summary

Nature Research wishes to improve the reproducibility of the work that we publish. This form provides structure for consistency and transparency in reporting. For further information on Nature Research policies, see our Editorial Policies and the Editorial Policy Checklist.

## Statistics

For all statistical analyses, confirm that the following items are present in the figure legend, table legend, main text, or Methods section.

| n/a | Confirmed | |
|---|---|---|
| ☐ | ☒ | The exact sample size (*n*) for each experimental group/condition, given as a discrete number and unit of measurement |
| ☐ | ☒ | A statement on whether measurements were taken from distinct samples or whether the same sample was measured repeatedly |
| ☐ | ☒ | The statistical test(s) used AND whether they are one- or two-sided<br>*Only common tests should be described solely by name; describe more complex techniques in the Methods section.* |
| ☐ | ☒ | A description of all covariates tested |
| ☐ | ☒ | A description of any assumptions or corrections, such as tests of normality and adjustment for multiple comparisons |
| ☐ | ☒ | A full description of the statistical parameters including central tendency (e.g. means) or other basic estimates (e.g. regression coefficient) AND variation (e.g. standard deviation) or associated estimates of uncertainty (e.g. confidence intervals) |
| ☐ | ☒ | For null hypothesis testing, the test statistic (e.g. $F$, $t$, $r$) with confidence intervals, effect sizes, degrees of freedom and $P$ value noted<br>*Give P values as exact values whenever suitable.* |
| ☒ | ☐ | For Bayesian analysis, information on the choice of priors and Markov chain Monte Carlo settings |
| ☒ | ☐ | For hierarchical and complex designs, identification of the appropriate level for tests and full reporting of outcomes |
| ☒ | ☐ | Estimates of effect sizes (e.g. Cohen's $d$, Pearson's $r$), indicating how they were calculated |

*Our web collection on statistics for biologists contains articles on many of the points above.*

## Software and code

Policy information about availability of computer code

| | |
|---|---|
| Data collection | Data collection details can be found at http://www.nurseshealthstudy.org. |
| Data analysis | Analyses were conducted with SAS version 9 (version 9.2, SAS Institute, Cary, NC, USA). Details can be found in the manuscript. |

For manuscripts utilizing custom algorithms or software that are central to the research but not yet described in published literature, software must be made available to editors and reviewers. We strongly encourage code deposition in a community repository (e.g. GitHub). See the Nature Research guidelines for submitting code & software for further information.

## Data

Policy information about availability of data

All manuscripts must include a data availability statement. This statement should provide the following information, where applicable:

- Accession codes, unique identifiers, or web links for publicly available datasets
- A list of figures that have associated raw data
- A description of any restrictions on data availability

The data that support the findings of this study are available from the Nurses' Health Studies but restrictions apply to the availability of these data, and so they are not publicly available. However, data are available from the authors upon reasonable request and with permission of Nurses' Health Studies External Advisory Board. Additional data sharing information and policy details can be accessed at http://www.nurseshealthstudy.org/researchers.

# Field-specific reporting

Please select the one below that is the best fit for your research. If you are not sure, read the appropriate sections before making your selection.

☒ Life sciences ☐ Behavioural & social sciences ☐ Ecological, evolutionary & environmental sciences

For a reference copy of the document with all sections, see nature.com/documents/nr-reporting-summary-flat.pdf

# Life sciences study design

All studies must disclose on these points even when the disclosure is negative.

| | |
|---|---|
| Sample size | A total of 488 cases and 1908 controls, in whom the original slides had been reviewed, were eligible for this study. Cases were women with biopsy-confirmed BBD who reported a subsequent diagnosis of breast cancer following their BBD diagnosis. Cases (invasive, in situ) were diagnosed between 1976 and 1998 for the NHS and between 1989 and 1999 for the NHS II. Eligible controls were women who completed the questionnaire in the same year that the breast cancer case was reported and had a previous diagnosis of biopsy-confirmed BBD but were free from breast cancer at the time of the case was diagnosed (index date; date of which the case was diagnosed). |
| Data exclusions | Women were excluded if they had evidence of in situ or invasive carcinoma at biopsy or reported a diagnosis of breast cancer within 6 months of their BBD biopsy. Women were excluded if they had unknown BBD lesion type. |
| Replication | Up to four controls were selected for each breast cancer case, matched on year of birth and year of BBD biopsy. |
| Randomization | Not applicable. |
| Blinding | Not applicable. |

# Reporting for specific materials, systems and methods

We require information from authors about some types of materials, experimental systems and methods used in many studies. Here, indicate whether each material, system or method listed is relevant to your study. If you are not sure if a list item applies to your research, read the appropriate section before selecting a response.

## Materials & experimental systems

| n/a | Involved in the study | |
|---|---|---|
| ☒ | ☐ | Antibodies |
| ☒ | ☐ | Eukaryotic cell lines |
| ☒ | ☐ | Palaeontology and archaeology |
| ☒ | ☐ | Animals and other organisms |
| ☐ | ☒ | Human research participants |
| ☒ | ☐ | Clinical data |
| ☒ | ☐ | Dual use research of concern |

## Methods

| n/a | Involved in the study | |
|---|---|---|
| ☒ | ☐ | ChIP-seq |
| ☒ | ☐ | Flow cytometry |
| ☒ | ☐ | MRI-based neuroimaging |

## Human research participants

Policy information about studies involving human research participants

| | |
|---|---|
| Population characteristics | This study is a nested case-control study within the Nurses' Health Study (NHS) and Nurses' Health Study II (NHS II) cohorts with biopsy-confirmed BBD. The NHS is an ongoing prospective cohort study that began in 1976, when 121,700 female registered nurses between the ages of 30 and 55 years completed a mailed questionnaire. The NHS II consists of 116,609 female registered nurses who were between the ages of 25 and 42 years when the study began in 1989. More details can be found at http://www.nurseshealthstudy.org/ . |
| Recruitment | Details can be found at http://www.nurseshealthstudy.org/ . |
| Ethics oversight | The study protocol was approved by the institutional review boards of the Brigham and Women's Hospital and Harvard T.H. Chan School of Public Health, and those of participating registries as required. Completion of the self-administered questionnaire was presumed to imply informed consent. |

Note that full information on the approval of the study protocol must also be provided in the manuscript.
